# Relationship Between Diet Quality and Glucose-Lowering Medication Intensity Among Adults With Type 2 Diabetes: Results From the CARTaGENE Cohort

**DOI:** 10.1016/j.cjco.2023.09.015

**Published:** 2023-09-29

**Authors:** Clémence Desjardins, Lise Leblay, Amélie Bélanger, Mathieu Filiatrault, Olivier Barbier, Line Guénette, Jacinthe Leclerc, Jean Lefebvre, Arsène Zongo, Jean-Philippe Drouin-Chartier

**Affiliations:** a Nutrition, Health and Society (NUTRISS) Research Center, Institute of Nutrition and Functional Foods (INAF), Laval University, Québec City, Québec, Canada; b Faculty of Pharmacy, Laval University, Québec City, Québec, Canada; c CHU de Québec-Université Laval Research Center, Québec City, Québec, Canada; d Research Center, Institut universitaire de cardiologie et de pneumologie de Québec-Université Laval, Québec City, Québec, Canada

## Abstract

**Background:**

In real-world settings, whether diet and medication are used as complements for glycemic management in type 2 diabetes (T2D) remains unclear. This study assessed the relationship between diet quality and intensity of glucose-lowering medication among adults with T2D.

**Methods:**

This cross-sectional study included 352 adults with T2D from the CARTaGENE Québec population-based cohort. Diet quality was assessed using the **H**ealthful **P**lant-Based **D**iet **I**ndex (hPDI). Glucose-lowering medication intensity was graded according to self-reported information on the type and number of drugs: no medication; oral monotherapy; oral polytherapy; and insulin with and without oral medication. In the subsample of 239 individuals who reported the medication dosages, intensity was also graded using the Medication Effect Score (MES).

**Results:**

In multivariable-adjusted models, we found no evidence of a relationship between the hPDI and medication intensity, assessed using the categorical approach (*P*_between-group_ = 0.25) or the MES (*P* = 0.43). However, the hPDI was inversely associated with the MES among men < 50 years of age and women < 60 years (β_1-point MES_ = –2.24 [95% confidence interval, –4.46, –0.02] hPDI points), but not among older individuals (β = –0.03 [–1.28, 1.21] hPDI points). Evidence of a nonsignificant inverse relationship between the hPDI and HbA1c was observed (β_10-point hPDI_ = –0.23% [–0.63, 0.17]), whereas a positive and significant association between the MES and hemoglobin (Hb)A1c was found (β_1-point MES_ = 0.30% [0.10, 0.51]).

**Conclusions:**

In this cohort of adults with T2D, there was an overall lack of complementarity between diet quality and intensity of glucose-lowering medication. The issue was particularly important among younger adults for whom diet quality was inversely associated with intensity of medication.

## Résumé

**Introduction:**

Il n'a jamais été étudié, en contexte réel, si la qualité de l'alimentation et la médication sont utilisées de façon complémentaire dans la gestion de la glycémie chez des personnes avec le diabète de type 2 (DT2). La présente a évalué la relation entre la qualité de l'alimentation et l’intensité de la médication hypoglycémiante chez des adultes avec DT2.

**Méthodes:**

Cette étude transversale portait sur 352 adultes avec DT2 participant à la cohorte populationnelle québécoise CARTaGENE. Nous avons évalué la qualité du régime à l’aide du **H**ealthful **P**lant-based **D**iet **I**ndex (hPDI, soit l’indice d’un régime alimentaire à base de plantes). Nous avons évalué l’intensité des médicaments hypoglycémiants à partir des renseignements fournis sur le type et le nombre de médicaments : aucun médicament, monothérapie orale, polythérapie orale, insuline avec ou sans médicaments par voie orale. Dans le sous-échantillon de 239 individus qui ont mentionné les posologies de médicaments, nous avons aussi évalué l’intensité au moyen du Medication Effect Score (MES).

**Résultats:**

Dans les modèles multivariés, nous n’avons observé aucune évidence de relation entre le hPDI et l’intensité des médicaments au moyen de l’approche catégorielle (*P*_entre les groupes_ = 0,25) ou du MES (*P* = 0,43). Toutefois, le hPDI était inversement associé au MES chez les hommes < 50 ans et chez les femmes < 60 ans (β_MES 1 point_ = –2,24 [intervalle de confiance à 95 %, –4,46, –0,02] points hPDI), mais non chez les personnes plus âgées (β = –0,03 [–1,28, 1,21] point hPDI). Nous avons observé une relation inverse non significative entre le hPDI et l’hémoglobine (Hb)A1c (β_hPDI 10 points_ = –0,23 % [–0,63, 0,17]) et une association positive et significative entre le MES et l’HbA1c (β_MES 1 point_ = 0,30 % [0,10, 0,51]).

**Conclusions:**

Au sein de cette cohorte d’adultes avec DT2, nous avons constaté une absence globale de complémentarité entre la qualité de l'alimentation et l’intensité des médicaments hypoglycémiants. Cet enjeu était d’autant plus important chez les personnes plus jeunes pour lesquels la qualité du régime était inversement associée à l’intensité des médicaments.

Type 2 diabetes (T2D) increases the risk of several macro- and microvascular diseases, including cardiovascular and renal diseases.^1^ T2D affects more than 500 million people worldwide, and this number is expected to exceed 700 million by 2045.^2^ Likewise, the global economic burden of T2D is on the rise and should exceed $2.5 trillion (USD) by 2030.^3^ As such, beyond prevention of T2D, its optimal management is a public health concern.

The cornerstone of T2D management relies on normalizing glycemic control. To do so, the complementary use of a healthy lifestyle—of which diet is a key component—and medication is warranted.^4^^,^^5^ Transitioning from a diet rich in ultraprocessed foods to a dietary pattern that emphasizes high intake of minimally processed plant foods and low intake of animal-based and ultraprocessed foods can reduce glycated hemoglobin (HbA1c) by 1.0% to 2.0%.^4^ In addition, such dietary pattern can also improve several risk factors for diabetes-related complications: namely, abdominal obesity, blood pressure, and lipid profile.^4^ In fact, optimal nutritional management can reduce glucose-lowering medication intensity and induce T2D remission.^4^^,^^6^ Glucose-lowering medication should be initiated upon diagnosis if HbA1c is judged too high or after 3 to 6 months of inconclusive attempts at normalizing glycemia with lifestyle modification.^5^ Metformin, the recommended first-line oral therapy, can alone reduce HbA1c by 0.5% to 1.5%.^5^ If insufficient, a plethora of glucose-lowering agents can be combined with or replace metformin to optimize glycemic management while also reducing the risk of macro- and microvascular complications.^5^

Adherence to dietary and pharmacologic therapies faces individual,^7^^,^^8^ social,^9^^,^^10^ and systemic barriers.^11^^,^^12^ However, little is known about how medication use influences diet quality. In the management of dyslipidemia and hypertension, 2 conditions closely related to T2D, observational studies reported that initiating medication could impede dietary modification and even prompt unfavourable dietary habits, likely because of the perception that medication is more effective and easier to implement than dietary changes.^13^, ^14^, ^15^, ^16^ Still, suboptimal glycemic management caused by the substitution of medication for diet is likely to lead to increased intensity of medication: thus, higher risks of side effects and medication nonadherence. Documenting the adequacy between dietary and pharmacologic management of T2D is of particular interest, as it has been reported that, among Canadian adults with T2D, approximately 50% have suboptimal glycemic management, and only one-third received recommendations on dietary changes from their primary care physicians.^17^

The objective of this study was to investigate the relationship between diet quality and intensity of glucose-lowering medication among adults with T2D from the Province of Québec, Canada. Specifically, we first evaluated the relationship between diet quality and intensity of glucose-lowering medication and explored individual characteristics modulating this relationship. We also explored how these 2 modalities were associated with plasma glycemic parameters.

## Methods

The protocol was reviewed and approved by the Comité d'éthique de la recherche en sciences de la santé de l'Université Laval and by CARTaGENE Sample and Data Access Committee.

### Study design and population

This study is a cross-sectional analysis within the CARTaGENE cohort. CARTaGENE is both a population-based biobank and a prospective cohort study in the Province of Québec, Canada.^18^ At the study’s inception, 43,037 Québec residents aged between 40 and 69 years were recruited. Recruitment took place during 2 phases (A: 2009-2010, n = 19,068; B: 2013-2014, n = 23,969). Participants were randomly selected from provincial health insurance registries to be representative of the Québec population based on age, sex, and area of residence. Individuals living in 6 metropolitan regions, where 70% of the Québec population lived, according to the 2006 census data (Montréal, Québec, Saguenay, Sherbrooke, Gatineau, Trois-Rivières), were invited to participate. CARTaGENE adhered to the Declaration of Helsinki, and all participants signed informed consent forms at the time of inclusion in the study.

The current analysis leverages data from phase A only, as solely participants from this phase were invited to complete a food frequency questionnaire (FFQ). During phase A, participants had to complete a health questionnaire during an in-person interview.^18^ The questionnaire included items on personal and family history of diseases (eg, diabetes, dyslipidemia, cancer), lifestyle (eg, alcohol, physical activity, sleep), and medication use (types and doses).^19^ Physical measures (eg, weight, height, waist circumference, blood pressure), as well as biological samples (eg, plasma, DNA) were collected during the interview. In 2012, phase A participants were invited by mail to complete the Canadian Dietary History Questionnaire II (C-DHQ II), a validated FFQ, which ended up being returned by ∼10,000 individuals.

For the current study, inclusion criteria were having self-reported a diagnosis of T2D (kappa [κ] for agreement between self-reported T2D diagnosis and report of T2D diagnosis in Québec administrative health database in CARTaGENE = 0.86)^20^; having adequately completed the FFQ (ie, < 40% of blank items); having reported plausible energy intakes (ie, women: 500-3500 kcal per day; men: 800-4200 kcal per day)^21^; and having provided a blood sample from which HbA1c was measured. Individuals with histories of cardiovascular disease, cancer, or kidney disease were excluded. A total of 352 adults with T2D were included in the study (Supplemental Fig. S1).

### Assessment of diet and diet quality

Diet was assessed using the C-DHQ II.^22^, ^23^, ^24^, ^25^ This FFQ, initially developed and validated by the US National Cancer Institute, was modified to reflect food availability, brand names, nutrition composition, and food fortification in Canada based on analyses of 24-hour dietary recalls reported by adults surveyed in the 2004 Canadian Community Health Survey.^26^ This FFQ assesses dietary intakes in the 12 months preceding its completion, addressing the frequency of consumption of 153 foods and the portion size usually consumed.

Diet quality was graded using the **H**ealthful **P**lant-Based **D**iet **I**ndex (hPDI), calculated from C-DHQ II data.^27^ The hPDI emphasizes high intakes of minimally processed plant foods,and low intakes of animal foods as well as processed and starchy plant foods. The decision of using the hPDI as the diet quality metric in this study was motivated by the fact that its calculation relies on intake of foods only—which facilitates knowledge translation—and because of its alignment with dietary recommendations for management of T2D and Canada’s food guide principles.^4^^,^^28^ The hPDI has been associated with lower risks of T2D, cardiovascular diseases, and mortality.^29^, ^30^, ^31^

The hPDI is calculated using intakes of 18 food groups, distributed into 3 categories: healthful plant-based foods, unhealthful plant-based foods, and animal-based foods. Healthful plant-based foods include whole grains, whole fruits, whole vegetables, nuts, legumes, vegetable oils, tea and coffee; unhealthful plant-based foods include fruit juices, refined grains, potatoes, sugar-sweetened beverages, sweets and desserts; and animal-based foods include animal fats, dairy products, eggs, fish and seafood, meat, and miscellaneous animal-based foods. For healthful plant-based foods, the score attributed to each participant was equivalent to its quintile of intake ranking. Unhealthful plant-based foods and animal-based foods were scored the opposite way. The subscores from each food group were then summed to obtain the overall hPDI score, ranging from 18 to 90. Higher scores reflect higher diet quality. The score was calculated separately for women and men.

### Assessment of medication

Information on medication was reported during the in-person interview.^18^ Participants were asked to bring all currently active prescribed medications (containers or a list of medications whenever possible) for the interviewer to record the product name based on the container labels.^32^ Agreement between reported medication and claim prescription records has been evaluated in a previous study and was considered excellent (κ > 0.80) for drugs used long term.^32^

In the current study, the intensity of glucose-lowering medication was graded according to the type and the number of medications, in line with clinical guidelines^5^: no medication; oral monotherapy; oral polytherapy; and insulin with or without oral medication. In secondary analyses, we graded the intensity of glucose-lowering therapy using the Medication Effect Score (MES).^33^ This score considers the number, types, dosages and expected reduction in HbA1c of each glucose-lowering medication.^33^ A higher MES denotes a greater expected reduction in HbA1c.^33^ The MES was calculated among participants who reported both the types and dosages of their glucose-lowering therapy adequately (n = 239).

### Assessment of HbA1c and plasma glucose

HbA1c and glucose were measured from the fasting plasma sample collected during the in-person interview. Upon collection, samples were sent to clinical diagnostic laboratories for immediate hematologic and biochemical analysis. Quality assurance tests demonstrated that all biochemical parameters were measured with test-retest reliability > 90%.^18^

### Assessment of covariables

Participants’ height was measured twice with a SECA 214 portable stadiometer (SECA, Hamburg, Germany), and a digital scale was used to measure their weight.^18^ Body mass index (BMI) was calculated from weight and height. The International Physical Activity Questionnaire was used to assess physical activity.^34^ Information on tobacco smoking was self-reported. Information on alcohol consumption (grams per day) was derived from the C-DHQ II.

### Statistical analyses

Statistical analyses were performed using SAS software, version 9.4 (SAS Institute, Cary, North Carolina, USA). All statistical tests were 2-sided, with a significance threshold set at *P* < 0.05. Missing data of covariables were imputed using the median or the most frequent category (Supplemental Table S1).

We first compared diet quality and dietary intakes according to intensity of glucose-lowering medication using linear regression models (GLM procedure). The hPDI was used as the dependent variable for analyses on diet quality. For dietary intakes, hPDI subscores were used as dependent variables to assess whether intakes of specific food groups differed according to intensity of glucose-lowering medication. Medication intensity was modelled as a categorical variable (no medication; oral monotherapy; oral polytherapy; and insulin with or without oral medication). In these analyses, Tukey-Kramer’s multiple comparison test was used to identify between-group statistical differences. Models were adjusted for gender (women/men), age (years), annual household income in Canadian dollars (CAD)(< $10,000, $10,000-$24,999, $25,000-$49,999, $50,000-$74,999, $75,000-$99,999, $100,000-$149,999, $150,000-$199,999, > $200,000), smoking status (never/past/current), physical activity level (low/moderate/high), self-reported history of high blood pressure (no/yes), self-reported history of dyslipidemia (no/yes), body mass index (kg/m^2^), energy intake (kcal per day), and alcohol consumption (grams per day). A post hoc F test was used to compute achieved power of this analysis using GPower software, version 3.1 (GPower ApS, Hinnerup, Denmark). As sensitivity analyses, we further adjusted for self-reported duration of T2D, which restricted the sample to n = 152. Next, we explored whether the differences in the hPDI associated with intensity of glucose-lowering medication were related to prespecified participant characteristics—gender (women vs men); age (men < 50 years and women < 60 years vs men ≥ 50 years and women ≥ 60 years); education level (high school or less vs college or university); annual household income (< $50,000 vs ≥ $50,000); smoking status (never vs past vs current); obesity (BMI < 30 vs ≥ 30 kg/m^2^); self-reported history of high blood pressure or high blood cholesterol (none vs high blood pressure or high blood cholesterol vs high blood pressure and high blood cholesterol)—using interaction tests. Evidence of interactions was assessed using the *P* value of the cross-product term between intensity of glucose-lowering medication and the stratification variable. The sex/gender-specific approach in the age stratification reflects the thresholds associated with higher age-related risk of cardiovascular disease (CVD).^35^^,^^36^ As confirmatory analyses, we repeated all of these by modelling intensity of glucose-lowering medication continuously using the MES. This restricted the sample to only participants who adequately reported both the types and dosages of their glucose-lowering therapy (n = 239).

Finally, we assessed the relationship between intensity of glucose-lowering medication, the hPDI, and HbA1c (%) and plasma glucose (mmol/L), using linear regression models. The same covariable structure as described here was used. Analyses with intensity of medication as the main independent variable were adjusted for the hPDI, and vice versa. We conducted these analyses using sequentially categories of intensity of glucose-lowering medication and the MES. Again, a post hoc F test was used to compute achieved power using GPower software, version 3.1.

In all analyses, linearity, homoscedasticity and normality postulates were assessed using distribution of the residual values, and a Box-Cox transformation was applied when needed.

## Results

Table 1 presents characteristics of the 352 individuals with T2D included in the study, according to intensity of glucose-lowering medication. A total of 64 (18.2%) participants were not using glucose-lowering medication; 149 (42.3%) were treated with an oral monotherapy; 100 (28.4%) had oral polytherapy; and 39 (11.1%) were using insulin with or without oral glucose-lowering medication. Individuals not using medication were more likely to be women, whereas individuals using medication, independent of intensity, were more likely to be men. BMI, the prevalence of dyslipidemia and high blood pressure, and the MES were higher among individuals using higher-intensity medication. Conversely, alcohol consumption and tobacco smoking were lower among individuals using higher-intensity medication. As expected, metformin was the most frequently used glucose-lowering drug, independent of overall medication intensity.

**Table 1:** Characteristics of the 352 individuals with T2D included in the study according to glucose-lowering medication intensity∗

Characteristics	Glucose-lowering medication intensity			
	No medication	Oral monotherapy	Oral polytherapy	Insulin with or without oral medication
Participants, n (%)	64 (18.2)	149 (42.3)	100 (28.4)	39 (11.1)
Age, years	57.3 ± 7.4	58.4 ± 7.2	59.3 ± 6.6	59.2 ± 8.1
Sex/gender				
Female/women, n (%)	34 (53.1)	62 (41.6)	40 (40.0)	17 (43.6)
Male/men, n (%)	30 (46.9)	87 (58.4)	60 (60.0)	22 (56.4)
Duration of T2D, years†	7.2 ± 9.5	6.9 ± 5.9	11.1 ± 7.6	12.1 ± 9.3
Education level, n (%)				
High school or less	18 (28.1)	49 (32.9)	39 (39.0)	20 (51.3)
College	18 (28.1)	45 (30.2)	38 (38.0)	10 (25.6)
University	28 (43.8)	55 (36.9)	23 (23.0)	9 (23.1)
Annual household income, n (%)				
< $50,000	34 (53.1)	73 (49.0)	54 (54.0)	23 (59.0)
$50,000$-99,999	23 (35.9)	53 (35.6)	30 (30.0)	15 (38.5)
≥ $100,000	7 (10.9)	23 (15.4)	16 (16.0)	1 (2.6)
hPDI, points	54.6 ± 2.2	52.6 ± 1.9	53.9 ± 2.2	53.4 ± 2.9
Smoking status, n (%)				
Never	19 (29.7)	66 (44.3)	31 (31.0)	14 (35.9)
Past	30 (46.9)	64 (43.0)	50 (50.0)	21 (53.8)
Current	15 (23.4)	19 (12.8)	19 (19.0)	4 (10.3)
Physical activity level, n (%)				
Low	9 (14.1)	37 (24.8)	22 (22.0)	7 (17.9)
Moderate	29 (45.3)	67 (45.0)	43 (43.0)	23 (59.0)
High	26 (40.6)	45 (30.2)	35 (35.0)	9 (23.1)
Alcohol consumption (g/day)	8.0 ± 21.8	7.4 ± 10.8	6.5 ± 17.0	6.6 ± 10.9
History of dyslipidemia, n (%)	38 (59.4)	97 (65.1)	64 (64.0)	30 (76.9)
History of high blood pressure, n (%)	30 (46.9)	76 (51.0)	64 (64.0)	22 (56.4)
Body mass index, kg/m^2^	30.2 ± 6.4	31.0 ± 5.6	33.3 ± 7.1	34.2 ± 6.4
HbA1c, %	6.55 ± 0.42	6.65 ± 0.35	7.43 ± 0.39	7.87 ± 0.52
Total medication, n	3 ± 3	5 ± 3	7 ± 2	7 ± 3
Glucose-lowering medication(s), n	0	1	2	2 ± 1
Medication Effect Score‡	0	0.4 ± 0.2	1.1 ± 0.7	2.9 ± 0.3
Metformin, n (%)	0 (0.0)	135 (90.6)	92 (92.0)	33 (84.6)
Sulfonylurea, n (%)	0 (0.0)	11 (7.4)	81 (81.0)	11 (28.2)
DPP-4 inhibitors, n (%)	0 (0.0)	1 (0.7)	20 (20.0)	3 (7.7)
Meglitinides, n (%)	0 (0.0)	1 (0.7)	2 (2.0)	0 (0.0)
Thiazolidinediones, n (%)	0 (0.0)	1 (0.7)	22 (22.0)	2 (5.1)
Combination of metformin and thiazolidinediones, n (%)	0 (0.0)	0 (0.0)	1 (1.0)	0 (0.0)
Insulin, n (%)	0 (0.0)	0 (0.0)	0 (0.0)	39 (100)
Insulin monotherapy, n (%)	0 (0.0)	0 (0.0)	0 (0.0)	5 (12.8)
Insulin with at least 1 oral medication, n (%)	0 (0.0)	0 (0.0)	0 (0.0)	34 (87.2)

HbA1c, hemoglobin A1C; hPDI, Healthful Plant-based Diet Index; T2D, type 2 diabetes.

∗ Continuous variables are presented as means ± standard deviation. Categorical variables are presented as count (percent).

† Data on duration of T2D were available for n = 152 of 352 participants (no medication: n = 29; oral monotherapy: n = 60; oral polytherapy: n = 48; insulin with or without oral medication: n = 15).

‡ Data on the Medication Effect Score were available for n = 239 of 352 participants (no medication: n = 64; oral monotherapy: n = 95; oral polytherapy: n = 58; insulin with or without oral medication: n = 22).

**Figure 1:**
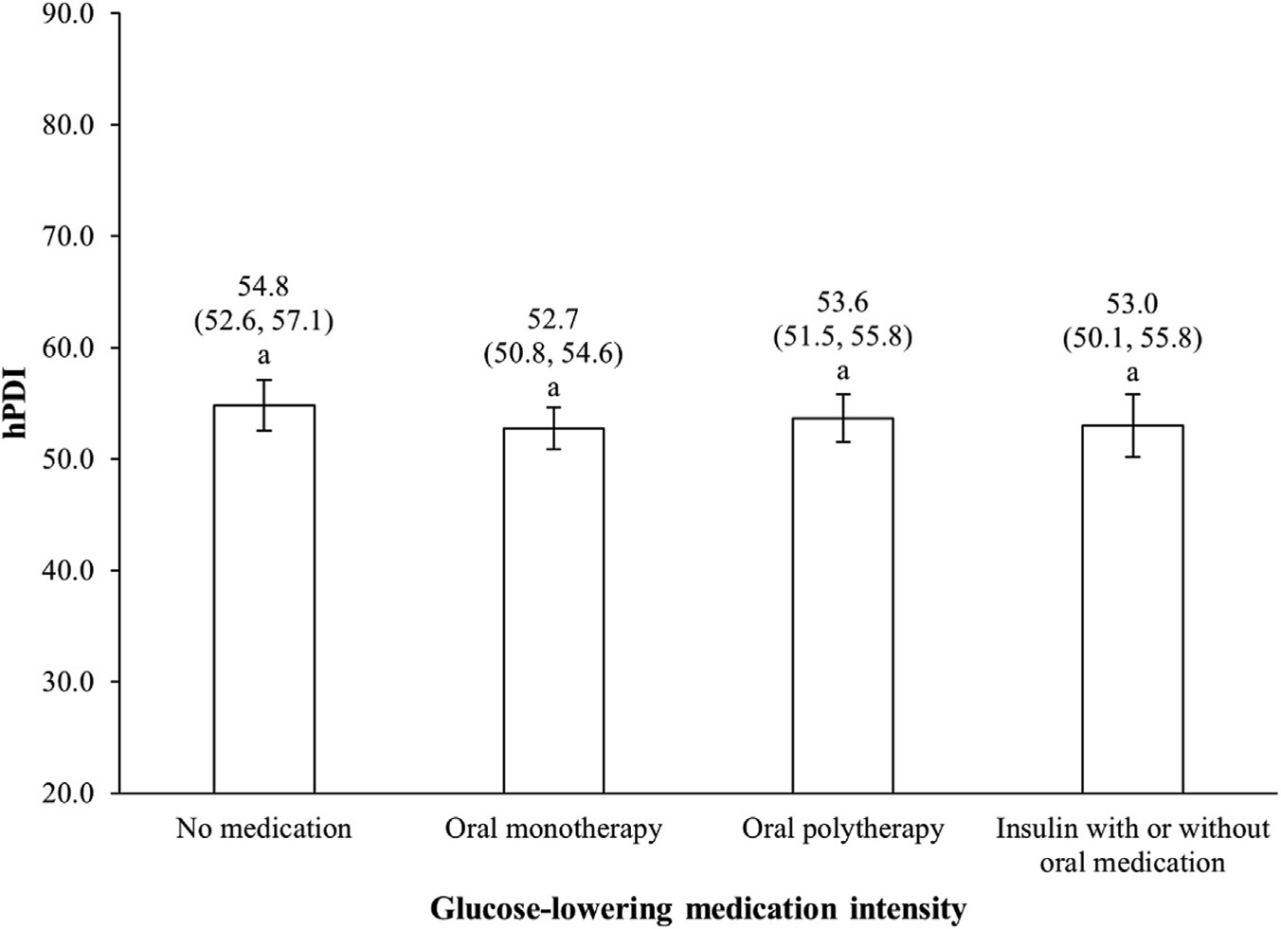
Healthful Plant-based Diet Index (hPDI) according to intensity of glucose-lowering medication. Data are presented as adjusted mean (95% confidence interval) following adjustment for gender, age, annual household income, body mass index, self-reported history of dyslipidemia, self-reported history of high blood pressure, physical activity level, smoking status, alcohol consumption, and energy intake. *P* value for between-group difference = 0.25. Columns with different letters are statistically different (*P* < 0.05).

We found no evidence of a difference in the hPDI according to intensity of glucose-lowering medication (Fig. 1). This observation was not because of low statistical power, as achieved statistical power of this model was 0.99. Further adjustments for duration of T2D in the subsample of participants for which this information was available (n = 152) yielded similar results (P _medication intensity_ = 0.46; data not shown). Likewise, we found no evidence of an association between the MES and the hPDI (Supplemental Table S2). However, stratified analyses revealed potential differences in the relationship between diet and intensity of glucose-lowering medication associated with smoking status, age, annual household income, and self-reported history of high blood pressure or dyslipidemia. Using the categorical classification for intensity of glucose-lowering medication (Supplemental Table S3) among participants who were smoking at the moment of data collection, mean hPDI significantly differed between those using insulin and those having oral monotherapy. However, these differences were not corroborated when intensity of glucose-lowering medication was graded continuously using the MES (Table 2). Still, a statistical trend suggested that the relationship between the MES and the hPDI differed according to the age of participants. A significant inverse association was observed between the MES and the hPDI among men < 50 years and women < 60 years but not among older individuals. Likewise, the hPDI and the MES were inversely associated among participants with annual household incomes of ≥ $50,000 as well as among those without concomitant history of high blood pressure or dyslipidemia. However, in these 2 analyses, the *P* values for interaction were > 0.10.

**Table 2:** Relationship between the MES and the hPDI, after stratification by key characteristics∗

Stratification	Difference in the hPDI associated with each 1 point increment in the MES	*P* value	*P* value for interaction
Gender			
Women	–0.65 (–2.04, 0.74)	0.36	0.63
Men	–0.10 (–1.86, 1.66)	0.91	
Age			
Men: < 50 y; women: < 60 y	–2.24 (–4.46, –0.02)	0.05	0.08
Men: ≥ 50 y; women: ≥ 60 y	–0.03 (–1.28, 1.21)	0.96	
Education level			
High school or less	–0.02 (–1.57, 1.54)	0.98	0.45
College or university	–0.85 (–2.39, 0.68)	0.27	
Annual household income			
< $50,000	0.14 (–1.31, 1.59)	0.85	0.14
≥ $50,000	–1.42 (–2.98, 0.14)	0.07	
Body mass index			
< 30 kg/m^2^	0.19 (–1.65, 2.03)	0.84	0.36
≥ 30 kg/m^2^	–0.84 (–2.18, 0.49)	0.21	
Smoking status			
Never	0.25 (–1.53, 2.02)	0.78	0.56
Past	–0.99 (–2.48, 0.51)	0.19	
Current	–0.02 (–3.26, 3.23)	0.99	
Self-reported history of high BP or dyslipidemia			
None	–4.00 (–7.96, –0.05)	0.05	0.21
High BP or dyslipidemia	–0.38 (–2.40, 1.63)	0.71	
High BP and dyslipidemia	–0.26 (–1.60, 1.08)	0.71	

BP, blood pressure; hPDI, Healthful Plant-Based Diet Index; MES, Medication Effect Score.

Data are presented as beta coefficient (95% confidence interval) after adjustment for sex/gender, age, annual household income, body mass index, self-reported history of dyslipidemia, self-reported history of high blood pressure, physical activity level, smoking status, alcohol consumption, and energy intake.

∗ Analyses included n = 239 participants who adequately reported both types and dosages of glucose-lowering medication they used, which allowed to calculate the Medication Effect Score.

With regard to dietary components of the hPDI, a statistical trend suggested that the subscore associated with whole grain consumption was lower among individuals using higher-intensity glucose-lowering medication (Table 3). When these analyses were repeated using the MES score, a statistical trend suggesting a positive association between the MES and subscore associated with intake of fruit juice (higher score reflects lower intakes) was observed (Supplemental Table S2).

**Table 3:** Healthful plant-based diet index subscores according to antidiabetic medication intensity∗

Dietary components	Glucose-lowering medication intensity				*P* value
	No medication	Oral monotherapy	Oral polytherapy	Insulin with or without oral medication	
Healthful plant-based foods	21.7 (20.2, 23.1)	20.5 (19.3, 21.7)	21.2 (19.8, 22.6)	20.0 (18.2, 21.9)	0.20
Whole grains	3.51 (3.07, 3.96)	3.05 (2.68, 3.42)	3.32 (2.90, 3.73)	2.97 (2.42, 3.52)	0.09
Vegetables	3.18 (2.78, 3.58)	2.94 (2.61, 3.27)	2.97 (2.59, 3.34)	2.78 (2.28, 3.28)	0.44
Fruits	2.86 (2.44, 3.28)	2.80 (2.45, 3.15)	2.86 (2.47, 3.25)	2.62 (2.10, 3.14)	0.73
Nuts	3.15 (2.71, 3.59)	3.08 (2.72, 3.45)	3.09 (2.68, 3.50)	2.98 (2.43, 3.52)	0.94
Legumes	3.08 (2.65, 3.51)	2.98 (2.62, 3.34)	3.06 (2.66, 3.46)	3.03 (2.50, 3.56)	0.94
Vegetable oils	2.83 (2.45, 3.22)	2.86 (2.54, 3.18)	2.99 (2.63, 3.35)	2.50 (2.02, 2.98)	0.16
Tea and coffee	3.04 (2.59, 3.48)	2.78 (50.7, 3.16)	2.92 (2.50, 3.33)	3.14 (2.58, 3.70)	0.47
Unhealthful plant-based foods	15.1 (14.1, 16.1)	14.3 (13.5, 15.2)	14.3 (13.4, 15.2)	14.6 (13.4, 15.9)	0.35
Fruit juices	2.72 (2.27, 3.17)	2.74 (2.36, 3.12)	2.80 (2.37, 3.22)	3.15 (2.59, 3.72)	0.52
Refined grains	3.18 (2.79, 3.57)	2.99 (2.67, 3.31)	2.76 (2.41, 3.12)	3.02 (2.54, 3.50)	0.27
Potatoes	3.24 (2.81, 3.66)	2.89 (2.54, 3.24)	2.76 (2.36, 3.16)	2.94 (2.41, 3.47)	0.18
Sugar-sweetened beverages	2.77 (2.39, 3.14)	2.64 (2.32, 2.95)	2.81 (2.46, 3.16)	2.51 (2.04, 2.98)	0.48
Sweets and desserts	3.20 (2.77, 3.63)	3.08 (2.72, 3.44)	3.17 (2.77, 3.57)	3.02 (2.49, 3.56)	0.85
Animal-based foods	18.1 (16.7, 19.4)	17.9 (16.8, 19.0)	18.1 (16.9, 19.4)	18.3 (16.6, 20.0)	0.94
Animal fats	2.95 (2.57, 3.32)	2.80 (2.49, 3.12)	2.89 (2.54, 3.24)	2.61 (2.14, 3.08)	0.50
Dairy	3.06 (2.63, 3.50)	3.09 (2.72, 3.45)	2.97 (2.56, 3.37)	3.27 (2.73, 3.81)	0.70
Eggs	3.12 (2.69, 3.55)	3.09 (2.73, 3.45)	3.05 (2.65, 3.45)	3.11 (2.57, 3.65)	0.99
Fish and seafood	3.10 (2.67, 3.53)	2.98 (2.62, 3.34)	3.28 (2.88, 3.68)	3.50 (2.96, 4.03)	0.13
Meat	3.00 (2.64, 3.37)	3.11 (2.80, 3.41)	3.15 (2.80, 3.49)	3.22 (2.76, 3.68)	0.81
Other animal foods	2.84 (2.48, 3.20)	2.82 (2.51, 3.12)	2.79 (2.45, 3.13)	2.59 (2.14, 3.05)	0.79

HbA1c, hemoglobin A1C.

∗ Data are presented as mean (95% confidence interval) after adjustment for gender, age, annual household income, body mass index, self-reported history of dyslipidemia, self-reported history of high blood pressure, physical activity level, smoking status, alcohol consumption, and energy intake.

**Figure 2:**
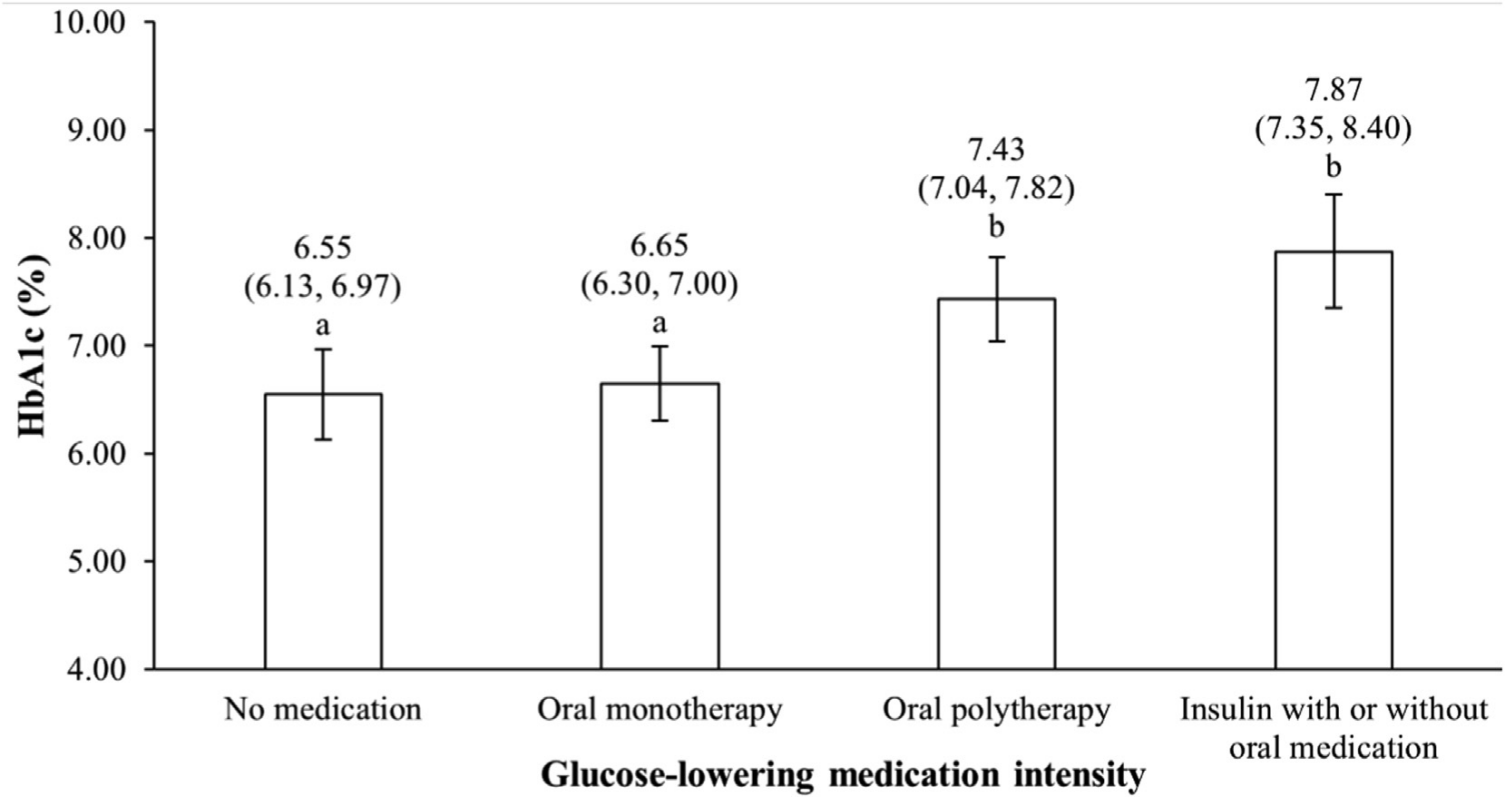
HbA1c (%) according to the intensity of glucose-lowering medication. Data are presented as mean (95% confidence interval) after adjustment for sex, age, body mass index, energy intake, Healthful Plant-Based Diet Index, physical activity level, annual household income, smoking status, alcohol consumption, self-reported history of dyslipidemia, and self-reported history high blood pressure. *P* value for between-group difference < 0.0001. Columns with different letters are statistically different (*P* < 0.05). HbA1c, hemoglobin A1c.

A significant inverse association between the crude hPDI and HbA1c and plasma glucose levels was observed in unadjusted analyses (Table 4). In multivariable-adjusted models, these associations were no longer statistically significant, even though the mean β coefficients remained negative. Per the post hoc F test, the achieved power of this model was 0.99. Individuals using insulin with or without concomitant oral glucose-lowering medication, as well as those with oral polytherapy had higher HbA1c than individuals with oral monotherapy or not using any glucose-lowering medication (Fig. 2). Analyses on plasma glucose revealed similar differences (Supplemental Fig. S2), as did analyses with further adjustment for duration of T2D (data not shown) or analyses on the relationship between the MES and HbA1c and plasma glucose (Supplemental Table S4).

**Table 4:** Relationship between the healthful plant-based diet index and glycemic parameters∗

Models†	HbA1c (%)		Plasma glucose (mmol/L)	
	β (95% CI)	*P* value	β (95% CI)	*P* value
Model 1	–0.21 (–0.42, –0.01)	0.03	–0.46 (–1.03, 0.11)	0.01
Model 2	–0.16 (–0.36, 0.04)	0.19	–0.32 (–0.89, 0.25)	0.09
Model 3	–0.23 (–0.63, 0.17)	0.16	0.01 (–1.08, 1.10)	0.42
Model 4	–0.16 (–0.40, 0.09)	0.14	–0.11 (–0.82, 0.61)	0.28

CI, confidence interval; HbA1c, hemoglobin A1c. T2D, type 2 diabetes.

∗ Data are presented as adjusted beta (95% confidence interval) associated with a 10-point increment in the healthful plant-based diet index.

† Model 1: Unadjusted (n = 352); Model 2: adjusted for sex, age, body mass index, energy intake, intensity of glucose-lowering medication, physical activity level, annual household income, smoking status, alcohol consumption, self-reported history of dyslipidemia, and self-reported history of high blood pressure (n = 352); Model 3: Model 2 + self-reported duration of T2D (n = 152); Model 4: same as Model 2, but intensity of glucose-lowering medication was replaced by the Medication Effect Score (n = 239).

## Discussion

In this cross-sectional analysis among adults with T2D from the CARTaGENE Québec population-based cohort, no evidence of complementarity between diet quality and intensity of medication for glycemic management was found in the overall sample. Actually, in younger individuals (men < 50 years and women < 60 years) and—potentially—among those with higher household incomes or who did not self-report concomitant dyslipidemia or high blood pressure, adherence to a healthy, plant-forward dietary pattern was negatively associated with intensity of glucose-lowering medication. These observations suggest that medication may be used as a substitute for diet quality in these subgroups of individuals with T2D. In addition, considering that, at the sample level, diet quality was suboptimal and that medication intensity was unfavourably associated with glycemic management, this study highlights the need for improved complementarity between diet quality and medication therapy in the management of T2D in Québec.

The lack of evidence of complementarity between diet quality and medication intensity in the management of T2D observed in this study needs to be interpreted in the context of the systemic, social, and individual challenges underlying the relationship between diet and medication use. From a systemic perspective, it is important to take into account that in Québec, over the first 2 decades of the 2000s, diet quality has remained both stable and suboptimal,^37^ whereas the prevalence of adults with T2D has increased by more than 40%.^38^ Also, over the past decade, 20% to 30% of the Québec population had no access to designated primary care providers.^39^ These statistics, when paralleled with our main observations, raise concerns with regard to the quality of T2D care at the provincial level. In the CARTaGENE cohort, no information on access to health professionals was collected, which impeded our ability to evaluate whether it influenced the diet-medication relationship. Still, the lack of evidence of a relationship between diet quality and intensity of medication observed at the sample level may reflect that medication was used to compensate for the inability to implement dietary modifications because of the numerous and multilevel barriers to healthy eating.^8^^,^^10^^,^^12^^,^^40^ Such an approach may be considered adequate at first sight. However, this observation was paralleled with a positive association between intensity of glucose-lowering medication and HbA1c levels, independent of duration of T2D. In fact, even when taking into account that individuals using sulfonylurea, meglitinides, or insulin may target less stringent HbA1c levels because of risk of hypoglycemia, most individuals treated with oral polytherapy or with insulin had HbA1c levels > 7.0%. Whether this association reflects concomitant pharmacotherapy adherence issues remains to be further assessed, but several qualitative studies reported that individuals with T2D experience important disease burdens and that part of it is attributable to the complexity of their medication regimen.^7^^,^^41^^,^^42^ Many individuals living with T2D consider that decreasing medication burden and achieving normoglycemia would have a substantial beneficial impact on their physical and emotional health.^7^^,^^41^ Thus, at the sample level, the lack of evidence of a complementary relationship between diet quality and intensity of medication is likely to fuel individual barriers to optimal diabetes care.

Stratified analyses allowed identification of subgroups among which not only was there no evidence of complementarity between diet quality and medication intensity but diet quality was negatively associated with intensity of medication. These observations could suggest that medication was used as a substitute for healthy dietary habits, which appears particularly problematic for long-term prevention of complications. The first subgroup among which we observed such relationship was composed of men < 50 years of age and women < 60 years of age. This finding is concerning, considering that younger individuals developing T2D live with the condition for a greater proportion of their lives, which increases the risk of complications independent of quality of glycemic management.^43^ From an individual perspective, the younger age of these individuals may bias their perception of long-term risks of complications and reduce the importance attributed to diet quality relative to medication.^44^^,^^45^ It is also plausible that individuals in this age group (ie, men < 50 years and women < 60 years) perceive medication as easier to use and manage than dietary changes, especially in the context that they are more likely to have burdening occupational responsibilities (eg, work, parenting) compared with older individuals. Finally, it cannot be excluded that this observation may also reflect different issues associated with the health care system. On the one hand, the perception of long-term risk of complications among health care professionals can also be biased, which may also alter importance attributed to diet in management of T2D.^46^, ^47^, ^48^ On the other hand, the negative relationship between diet quality and medication intensity may also reflect an overall lack of access to multidisciplinary health care services. Still, we stress that no information on access to health professionals was available, which impeded our ability to evaluate whether it influenced the diet-medication relationship. For the 2 other subgroups among which the MES and the hPDI were inversely associated—individuals with higher household income and those without concomitant dyslipidemia or high blood pressure—we recognize that interaction tests did not meet the threshold for statistical significance. Still, these observations warrant discussion, as they also reflect potential substitution of medication for healthy dietary habits. The observation among individuals with annual household incomes of > $50,000 was unexpected, as individuals with higher socioeconomic status usually face more facilitators to healthy eating and medical care. However, the apparent lack of consideration for diet quality may be reflective of a facilitated financial access to drug therapies.^45^^,^^49^ If perceived as more effective and easier to implement than dietary changes, this may prompt unfavourable dietary habits.^16^^,^^49^ Finally, as for younger individuals, the negative relationship between diet quality and intensity of medication among individuals who had not self-reported histories of high blood pressure or dyslipidemia may also reflect low perception of risk among both the individuals with T2D and the health professionals.^46^, ^47^, ^48^

### Limitations and strengths

Results of this study need to be interpreted in the context of limitations and strengths. First, the main limitation of this work is related to the 2- to 3-year gap between medication and glucose homeostasis assessments (2009-2010) and the completion of the FFQ (2012). Indeed, we cannot exclude that medication or diet changed during this period. Similarly, because of the cross-sectional design, we could not assess the temporal dynamics between diet quality and intensity of medication. Still, we observed negative relationships between the hPDI and HbA1c and plasma glucose. Even though these did not reach statistical significance, it suggests that diet did not change significantly between medication and glucose homeostasis assessments (2009-2010) and the completion of the FFQ (2012). Also, because of the moment CARTaGENE data collection was conducted, no participant was using sodium-glucose cotransporter-2 inhibitors or glucagon-like peptide-1 receptor agonists, as these were not yet approved in Canada. However, we graded intensity of medication in both categorical and continuous fashions, which accurately depicted intensification approaches according to national guidelines for T2D.^5^ Therefore, even if medications were not as diversified as they are now, we can still expect conclusions to be similar. Finally, adherence to pharmacotherapy was not considered in this study. An analysis on the relationship between adherence to diet guidelines and glucose-lowering medication should be conducted in the future.

With regard to strengths, the post hoc F tests we conducted suggest that our analyses were sufficiently powered, further supporting a true lack of relationship between diet quality and intensity of glucose-lowering medication. On the other hand, this suggests that the lack of a statistically significant inverse relationship between the hPDI and HbA1c was not the result of a lack of statistical power. This observation may instead be attributed to the fact that the hPDI does not focus only on intake of foods that directly affect plasma glucose. In fact, of the 18 food groups considered in its calculation, the consumption of only 7 has a direct effect on plasma glucose (ie, whole grains, whole fruits, fruit juices, refined grains, potatoes, sugar-sweetened beverages, sweets, and desserts). Finally, this study addresses an issue that has never been previously assessed in Canada or Québec.

## Conclusions

In this sample of adults with T2D, there was an overall lack of complementarity between diet quality and intensity of medication for glycemic management. The issue was particularly important among younger adults for whom diet quality was inversely associated with intensity of glucose-lowering medication. Furthermore, intensity of glucose-lowering medication was unfavourably associated with glycemic management. This study highlights the need for improved dietary and interdisciplinary care to optimize the complementarity between diet quality and medication for glucose management in people living with T2D in Québec.

## Data Availability

Data described in the manuscript, code book, and analytic code will not be made publicly available. Additional information on the procedures for obtaining and accessing data from the CARTaGENE cohort is described at https://cartagene.qc.ca/.
