# Supplementary material for: Relationship Between Diet Quality and Glucose-Lowering Medication Intensity Among Adults With Type 2 Diabetes: Results From the CARTaGENE Cohort
Source: CJC Open. 2023 Sep 29;6(1):20–9. doi: 10.1016/j.cjco.2023.09.015 (PMC10837702; doi:10.1016/j.cjco.2023.09.015)
Supplement: Supplemental Tables and Figures [file mmc1.docx]

**Supplemental material**

**Relationship between diet quality and glucose-lowering medication intensity among adults with type 2 diabetes: results from the CARTaGENE cohort.**

Clémence Desjardins, Lise Leblay, Amélie Bélanger, Mathieu Filiatrault, Olivier Barbier, Line Guénette, Jacinthe Leclerc, Jean Lefebvre, Arsène Zongo, Jean-Philippe Drouin-Chartier

**Corresponding author:**

Jean-Philippe Drouin-Chartier

[Jean-philippe.douin-chartier@pha.ulaval.ca](mailto:Jean-philippe.douin-chartier@pha.ulaval.ca)

**Supplemental Table S1: Covariables for which missing value were imputed.^1^**

| **Covariables** | **Imputed value** | **Imputations, n (%)** |
| --- | --- | --- |
| Body mass index, kg/m^2^ | 31.1 | 4 (1.1) |
| Physical activity level | Moderate | 25 (7.1) |
| Plasma glucose, mmol/L | 7.00 | 11 (3.1) |
| Annual household income, $ | $50,000-75,999 | 21 (6.0) |
| Smoking status | Never smoker | 1 (0.3) |
| Self-reported history of dyslipidemia | No dyslipidemia | 3 (0.9) |
| Self-reported history of high blood pressure | No high blood pressure | 2 (0.6) |
| Education level | High school | 2 (0.6) |

^1^ Missing values of continuous variables were imputed using the median. Missing values of categorical variables were imputed using the most frequent category.

**Supplemental Table S2: Differences in the healthful plant-based diet index (hPDI) and hPDI sub-scores associated with a 1-point increment in the medication effect score.^1^**

| **Dietary components** | **β (95% CI)** | ***P* value** |
| --- | --- | --- |
| Total hPDI score | -0.44 (-1.54, 0.66) | 0.43 |
| Healthful plant-based foods | -0.35 (-1.06, 0.36) | 0.33 |
| Whole grains | -0.13 (-0.34, 0.08) | 0.23 |
| Vegetables | -0.13 (-0.30, 0.05) | 0.17 |
| Fruits | -0.07 (-0.27, 0.14) | 0.53 |
| Nuts | -0.07 (-0.27, 0.13) | 0.51 |
| Legumes | 0.02 (-0.17, 0.22) | 0.81 |
| Vegetable oils | -0.09 (-0.27, 0.09) | 0.35 |
| Tea and coffee | 0.10 (-0.11, 0.31) | 0.36 |
| Unhealthful plant-based foods | 0.01 (-0.46, 0.49) | 0.96 |
| Fruit juices | 0.19 (-0.02, 0.40) | 0.08 |
| Refined grains | -0.03 (-0.21, 0.16) | 0.81 |
| Potatoes | -0.07 (-0.27, 0.12) | 0.47 |
| Sugar-sweetened beverages | 0.02 (-0.16, 0.20) | 0.84 |
| Sweets and desserts | -0.10 (-0.30, 0.11) | 0.36 |
| Animal-based foods | -0.10 (-0.71, 0.51) | 0.75 |
| Animal fats | -0.18 (-0.35, -0.01) | 0.03 |
| Dairy | 0.02 (-0.19, 0.22) | 0.88 |
| Eggs | 0.10 (-0.09, 0.30) | 0.30 |
| Fish and seafood | 0.08 (-0.12, 0.28) | 0.44 |
| Meat | -0.05 (-0.22, 0.12) | 0.54 |
| Other animal foods | -0.06 (-0.23, 0.10) | 0.45 |

^1^ These analyses were conducted among the 239/352 participants who adequately reported type and dosages of glucose-lowering medication, which allowed to calculate the medication effect score. Data are presented as adjusted beta (95% confidence interval) associated with a 1-point increment in the medication effect score. Models were adjusted for gender, age, annual household income, body mass index, self-reported history of dyslipidemia, self-reported history of high blood pressure, physical activity level, smoking status, alcohol consumption, and energy intake.

**Supplemental Table S3: Healthful plant-based diet index according to glucose-lowering medication intensity, following stratification for key characteristics.^1^**

| **Characteristics** | **No medication** | **Oral monotherapy** | **Oral polytherapy** | **Insulin with or without oral medication** | ***P* value for interaction** |
| --- | --- | --- | --- | --- | --- |
| Gender |  |  |  |  |  |
| Women | 54.5  (51.6, 57.4) | 52.6  (50.3, 55.0) | 53.3  (50.6, 56.1) | 53.7  (49.9, 57.5) | 0.91 |
| Men | 55.1  (52.2, 58.1) | 52.8  (50.6, 54.9) | 53.8  (51.3, 56.3) | 52.3  (48.8, 55.9) |  |
| Age |  |  |  |  |  |
| Male/men: <50 y; Female/women: <60 y | 55.4  (52.5, 58.4) | 51.8  (49.4, 54.3) | 53.8  (50.7, 56.9) | 50.3  (45.4, 55.1) | 0.47 |
| Male/men: ≥50 y; Female/women: ≥60 y | 54.6  (51.8, 57.4) | 53.3  (51.2, 55.4) | 53.7  (51.3, 56.0) | 53.9  (50.8, 57.0) |  |
| Education level |  |  |  |  |  |
| High school or less | 53.6  (49.8, 57.4) | 53.0  (50.3, 55.6) | 53.1  (50.3, 55.9) | 53.5  (49.8, 57.2) | 0.72 |
| College or university | 55.3  (52.8, 57.8) | 52.6  (50.5, 54.6) | 53.9  (51.5, 56.3) | 52.3  (48.6, 55.9) |  |
| Annual household income |  |  |  |  |  |
| <50,000$ | 54.6  (52.1, 57.0) | 53.4  (51.7, 55.1) | 54.0  (52.0, 55.9) | 54.5  (51.5, 57.5) | 0.47 |
| ≥50,000$ | 55.5  (52.9, 58.2) | 52.1  (50.3, 53.8) | 53.0  (50.9, 55.1) | 50.9  (47.4, 54.5) |  |
| Body mass index |  |  |  |  |  |
| <30 kg/m^2^ | 55.1  (52.2, 58.0) | 53.6  (51.3, 55.9) | 54.3  (51.5, 57.0) | 56.0  (51.4, 60.6) | 0.57 |
| ≥30 kg/m^2^ | 54.8  (51.9, 57.7) | 52.1  (50.0, 54.3) | 52.7  (50.3, 55.2) | 51.3  (48.1, 54.4) |  |
| Smoking status |  |  |  |  |  |
| Never | 54.1  (51.0, 57.3) | 52.7  (51.0, 54.3) | 53.8  (51.4, 56.3) | 52.5  (48.8, 56.2) | 0.007 |
| Past | 54.9  (52.3, 57.4) | 53.8  (52.0, 55.6) | 54.0  (51.4, 55.9) | 51.1  (48.0, 54.1) |  |
| Current | 56.4  (52.7, 60.1)^ab^ | 49.6  (46.4, 52.7)^a^ | 52.5  (49.3, 55.7)^ab^ | 63.8  (57.0, 70.5)^b^ |  |
| Self-reported history of high blood pressure or dyslipidemia |  |  |  |  |  |
| None | 58.5  (54.5, 62.5) | 54.0  (51.0, 56.9) | 54.3  (50.6, 58.0) | 54.0  (51.0, 56.9) | 0.82 |
| High blood pressure or dyslipidemia | 53.5  (50.6, 56.5) | 52.4  (50.0, 54.8) | 52.5  (49.7, 55.2) | 52.4  (50.0, 54.8) |  |
| High blood pressure and dyslipidemia | 54.0  (50.6, 57.4) | 52.3  (50.0, 54.6) | 54.1  (51.5, 56.7) | 52.3  (50.0, 54.6) |  |

^1^ Data are presented as mean (95% confidence interval) after adjustment for sex/gender, age, annual household income, body mass index, self-reported history of dyslipidemia, self-reported history of high blood pressure, physical activity level, smoking status, alcohol consumption, and energy intake. Different letters denote statistically significant differences between groups (*P*<0.05).

**Supplemental Table S4: Relationship between the medication effect score and glycemic parameters.^1^**

| **Models^2^** | **HbA1c (%)** | | **Plasma glucose (mmol/L)** | |
| --- | --- | --- | --- | --- |
|  | **β (95% CI)** | ***P* value** | **β (95% CI)** | ***P* value** |
| Model 1 | 0.34  (0.15, 0.52) | <0.0001 | 0.89  (0.34, 1.44) | 0.0003 |
| Model 2 | 0.30  (0.10, 0.51) | <0.0001 | 0.83  (0.24, 1.42) | 0.009 |

^1^ Data are presented as adjusted Beta (95% confidence interval) associated with a 1-point increment in the medication effect score.

^2^ Model 1: unadjusted (n=239); Model 2: adjusted for sex, age, body mass index, energy intake, healthful plant-based diet index, physical activity level, annual household income, smoking status, alcohol consumption, self-reported history of dyslipidemia, and self-reported history of high blood pressure (n=239).


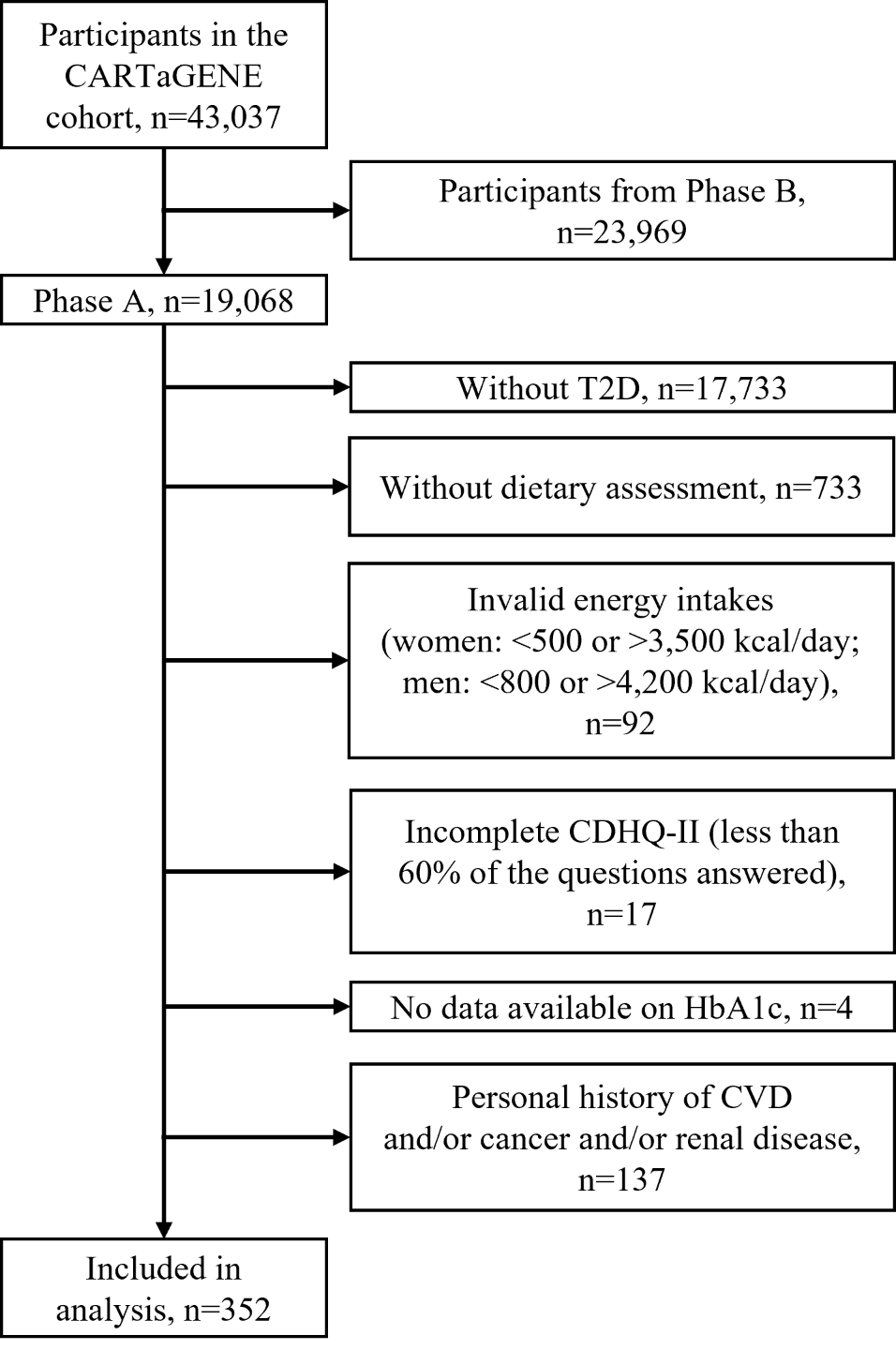


**Supplemental Figure S1: Flow chart of participant selection.**

**
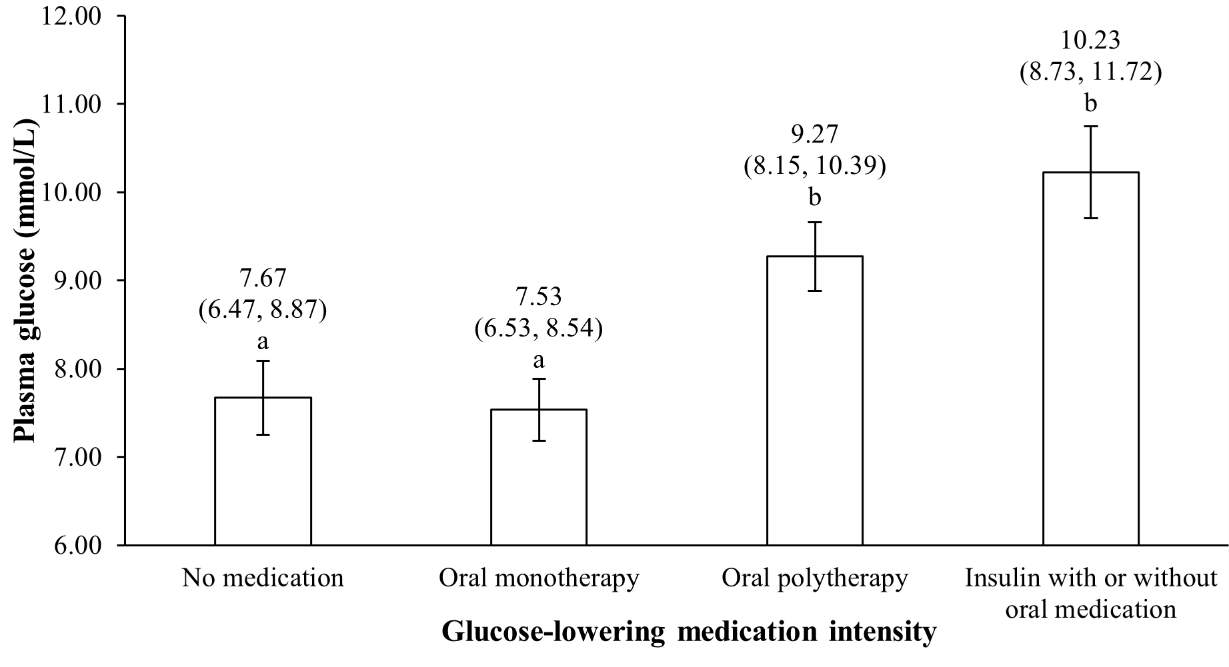
**

**Supplemental Figure S2: Plasma glucose (mmol/L) according to the intensity of glucose-lowering medication.** Data are presented as mean (95% confidence interval) after adjustment for sex, age, body mass index, energy intake, healthful plant-based diet index, physical activity level, annual household income, smoking status, alcohol consumption, self-reported history of dyslipidemia, and self-reported history high blood pressure. *P* value for between-group difference<0.0001. Columns with different letters are statistically different (*P*<0.05).
